# Anti-phospholipid antibodies in the setting of thromboembolic events associated with severe COVID-19 pneumonia

**DOI:** 10.1186/s43166-021-00105-x

**Published:** 2022-01-18

**Authors:** Omaima Ibrahim Badr, Wael Aly Elrefaey, Mohammed Hassan Abu-Zaid, Samah Hamdy Elmedany

**Affiliations:** 1 Department of Chest Medicine, Faculty of Medicine, Mansoura University, Dakahlia Governorate, Egypt; 2 Department of Chest Medicine, Al Noor Specialist Hospital, Mecca, Saudi Arabia; 3 Department of Rheumatology and Rehabilitation, Faculty of Medicine, Tanta University, El-Geish Street, Tanta, Gharbia Egypt; 4 Department of Rheumatology and Rehabilitation Medicine, Al Noor Specialist Hospital, Mecca, Saudi Arabia

**Keywords:** COVID-19, Severe pneumonia, Venous thromboembolism, Arterial thromboembolism, Anti-phospholipid antibodies

## Abstract

**Background:**

Thrombotic consequences have been reported in COVID-19-infected patients, especially those who are critically ill. Multiple studies have tested antiphospholipid antibodies (aPLs) among COVID-19 patients, but to date, the actual frequency of aPLs is still uncharted.

In this cohort study, we analyzed the outcomes of 173 consecutive patients with confirmed COVID-19 infection. Anti-phospholipid antibodies, which include anti-cardiolipin antibodies [aCL (IgM), aCL (IgG)], and B2-glycoprotein I antibodies [aβ2GPI (IgM), aβ2GPI (IgG)] were detected by using immunoassays. In contrast, lupus anti-coagulant (LAC) antibodies are identified through a coagulation-based assay.

**Results:**

The study demonstrated a high incidence of thrombotic consequences in severe COVID pneumonia cases and supported an increased risk of developing aPLs following COVID-19 infection. Pulmonary embolism had the most common prevalence of all thrombotic events. Among the various aPLs tested in thrombotic patients, lupus anti-coagulant (LAC) had the highest positivity (46.2%). Most patients with arterial thromboembolism (stroke, myocardial infarction, limb ischemia, bowel ischemia, and renal artery thrombosis) had triple positivity of anti-phospholipid antibodies. Testing aPLs antibodies after 12 weeks of recovery for survived patients only 2 out of 23 patients had aPLs positivity compared to 35 out of 65 tested during hospital admission. Furthermore, we found no significant changes in aPLs positivity between survived and non-survived patients with thrombotic event.

**Conclusions:**

aPLs increased transiently as an inflammatory-mediated condition. Individuals with aPLs triple positivity (positive LAC, aCL, and aB2GPI) had a considerable risk of arterial thromboembolism (ATE).

## Background

The coronavirus disease 2019 (COVID-19) caused by the severe acute respiratory syndrome coronavirus 2(SARS-CoV-2) is a major public health emergency in recent times [1]. Multi-organ failure secondary to infection by coronavirus has been labeled as the primary reason for mortality in COVID-19 [2, 3].

Recently, several trials have reported an exceptionally high prevalence of thrombotic events, suggesting that these complications may contribute to death [4, 5]. A variety of studies have revealed thromboembolic consequences, including venous thromboembolism (VTE) (pulmonary embolism (PE), and deep vein thrombosis (DVT)), as well as arterial thromboembolism (ATE), which includes (cerebral infarction, myocardial infarction, and limb arterial thrombosis). An autopsy of a COVID-19 victim revealed micro pulmonary thrombosis at a rate of up to 80% [6].

The crucial role of thrombo-inflammation and endothelial damage in thromboembolism has long been known [7]. The overproduction of IL-1, interleukin (IL-6), IL-8, and tumor necrosis factor (TNF), as a pro-inflammatory cytokine, is thought to be the cause of “cytokine release syndrome” or “cytokine storm.” In addition to pro-inflammatory marker increases, hypercoagulability has been recognized as a key factor in determining the prognosis of those patients [8]; however, the actual mechanism for thromboembolic complications is still unknown.

Anti-phospholipid antibodies (aPLs) are a class of antibodies that include the anti-cardiolipin (aCL), lupus anti-coagulant (LAC), and anti-B2-glycoprotein I (aB2GPI) antibodies, all of which have phospholipid-binding proteins as their principal targets. The relationship of viral infections with aPLs is described before in the literature. Individuals infected with viruses such as HIV, HCV, HBV, human T-lymphotropic virus type 1 (HTLV-1), Epstein-Barr virus (EBV), varicella virus, cytomegalovirus (CMV), parvovirus B19, streptococcal and staphylococcal infections, and gram-negative organisms are highly associated with aPLs positivity [9]**.** It postulates that by molecular mimicry, some of the infectious agents might induce nonpathogenic aPLs and pathogenic anti-β2-GPI [9].

Reports of appearing aPLs in COVID-19 patients and their putative relationship to thrombosis have started to emerge in case series and case reports [10, 11]. Anti-cardiolipin (aCL) and anti-B2-glycoprotein I (aB2GPI) antibodies were found in three critical COVID-19 patients having multiple cerebral infarctions in a previous study suggesting for the first time that COVID-19-related coagulopathy could be an acquired thrombophilia close to the spectrum of anti-phospholipid syndrome (APS) [11]. The major difference between APS and COVID-19-associated thrombosis is the normal fibrinogen levels in APS, which comes in contrast to COVID-19 thrombosis [11].

Multiple studies have tested aPLs antibodies among COVID-19 patients [11–14], but to date, the real frequency of aPLs is still uncharted. This study aimed to shed light on the association of aPLs and the development of thromboembolic events (arterial and venous) in severe COVID-19 pneumonia patients and whether these positive antibodies continue after improvement, as there is minimal evidence in the previous investigations. We also seek to identify the type of aPLs found in patients with COVID-19, as well as the possible association of these aPLs with other distinctive characteristics of COVID-19.

## Methods

### Study design

A prospective cohort study was conducted at Al-Noor Specialist, tertiary care institute, Makkah, Saudi Arabia. 

#### Inclusion and exclusion criteria

Between 21 October 2020 and 30 March 2021, all patients with severe COVID-19 pneumonia (SARS-CoV-2-infected individuals with SpO2 93% on room air, PaO2/FiO2 ratio less than 300 mmHg, rate of respiration > 30 breaths/minute, or pulmonary infiltrates > 50%) [15] with probable thromboembolic complications were included in our study. Patients under 18 years and those with a history of the anti-phospholipid syndrome were excluded.

### Data collection and study procedures

Data were obtained from medical files and electronic records using a distinctive medical record number (MRN). Demographic information of the patients (age, gender, nationality, and smoking history), as well as clinical symptoms (cough, fever, SOB, body aches, headache, nausea, vomiting, diarrhea, loss of taste, and loss of smell), comorbidities, and a chest radiograph, were gathered at the admission time to the hospital. Clinical indicators such as (respiration rate, heart rate, and oxygen saturation percent on room air, limb weakness, calf pain, and abdomen rigidity) were gathered at the time of suspicion of thromboembolic consequences.

Age, smoking history, obesity (defined as a BMI > 30), D-dimer level, length of hospital stays, comorbidities, disseminated intravascular coagulation (DIC) including CBC (WBC, platelets, and hemoglobin level) and coagulation parameters (PT, PTT, and INR) were all collected as part of the risk assessment for thromboembolism.

Suspected thromboembolic complications, either venous or arterial thromboembolism, were diagnosed in COVID-19 patients utilizing a Computed Tomography pulmonary angiogram (CTPA) to diagnose pulmonary embolism (PE). Doppler ultrasound for the diagnosis of vascular (arterial/venous) thrombosis, brain computed tomography (CT) to detect infarction, ECG and echocardiography to diagnose myocardial ischemia, and abdominal CT angiography for diagnosis of vascular thrombosis and mesenteric/bowel ischemia.

Anti-phospholipid antibodies, which include anti-cardiolipin antibodies [aCL (IgM), aCL (IgG)], and B2-glycoprotein I antibodies [aβ2GPI (IgM), aβ2GPI (IgG)] were detected by using immunoassays that measure reactivity to cardiolipin, a phospholipid, and b2-glycoprotein I, a phospholipid-binding protein, respectively. Lupus anti-coagulant (LAC) antibodies were identified through a coagulation-based assay that demonstrates prolongation of a phospholipid-dependent clotting time. The results of at least one anti-phospholipid antibody test were reported as being positive. The term “mono positivity” refers to one of the three aPLs (LAC, aCL, or a2GPI), “double positivity” refers to two of the three aPLs, and “triple positivity” refers to all three aPLs. All patients were given prophylactic anti-coagulants (subcutaneous fractionated or unfractionated heparin) during their hospital stay, according to the hospital VTE policy. Serum aPLs antibodies were retested after 12 weeks from the first sample for the previously positive survived cases with thrombotic events. All retested patients were discharged as outpatients, based on the COVID-19 hospital discharge criteria (afebrile for at least 24 h without anti-pyretics, improved respiratory symptoms such as cough and shortness of breath, and two negative specimens collected 24 h apart), and they were all on therapeutic oral anti-coagulants. The final date of follow-up was 10 July 2021.

### Statistical analysis plan

Statistical Package for Social Science (SPSS) version 22 was used in analyzing the collected information after it was recorded, coded, and tabulated using Windows on a personal computer. Patients’ demographic parameters, clinical signs and symptoms, comorbidities, and radiological findings were described using descriptive statistics. Kolmogorov-Smirnov test for normality was used to assess the distribution of continuous variables. Normally distributed continuous data were described as mean ± SD, and for data that were not normally distributed, median (interquartile range (IQR)) was used. Qualitative data were described as percentages (frequencies). The Mann–Whitney *U* test was used to compare the median value for non-normally distributed continuous variables. The independent sample *t* test was used to compare the mean value for normally distributed. A chi-squared test/Fisher test was used as appropriate to compare proportions for qualitative variables. Paired comparison of nominal data was done using McNemar test. A confidence interval of 95% (*p* < 0.05) was applied to characterize the statistical significance of the results, and the level of significance was assigned as 5%.

### Ethical part and confidentiality

The Saudi Arabian Ministry of 131 Health's institutional ethics board approved this study (No. H-02-K-076-0920-386).

## Results

**Fig. 1:**
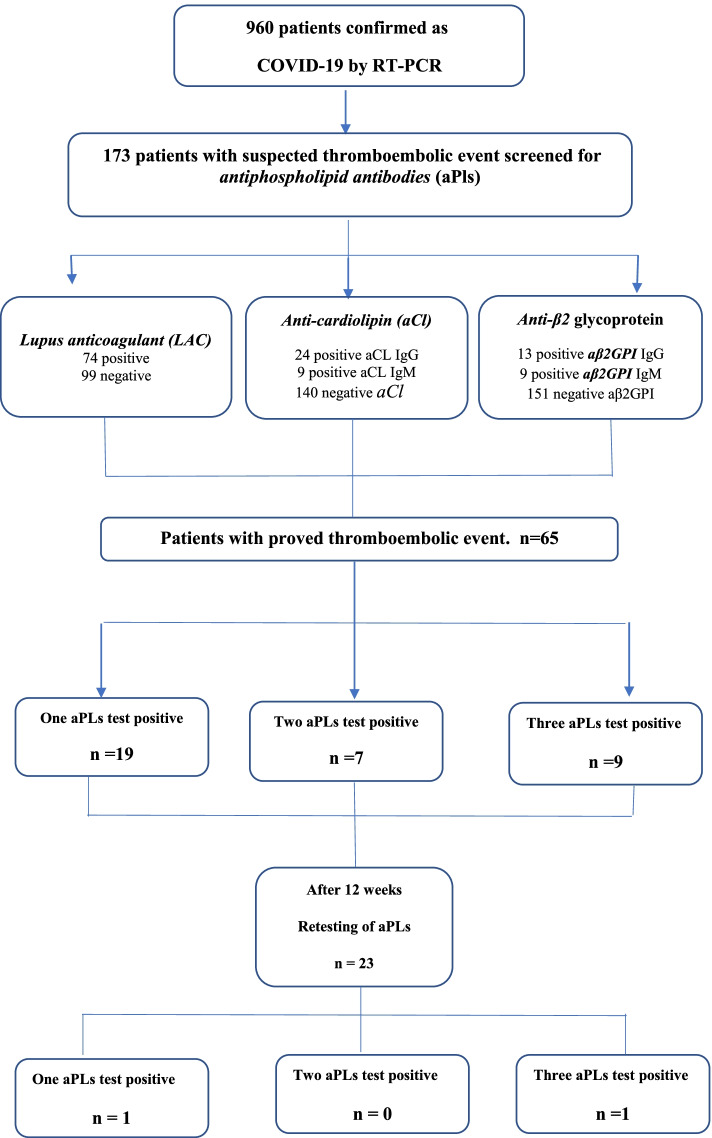
Flow chart of the study design

Among 960 admitted COVID-19 patients confirmed by a real-time polymerase chain reaction (PCR), 173 patients with severe pneumonia screened for thromboembolic complications, of which 65 patients had proven thromboembolic events. For the 65 patient aPLs were tested during hospitalization, 19 (29.2%) patients were mono positive, 7 (10.7%) were double-positive, and 9 (13.8%) had triple-positivity. Following re-testing for aPLs in 23 patients 12 weeks after the initial sample, only 2 patients tested positive for aPLs (one patient had single aPLs positivity and the other patient had three aPLs test positivity) (Fig. 1).

Venous thrombosis includes (PE, DVT, and PE with DVT) was detected in 53(30%) out of 173 patients; however, arterial thrombosis includes (stroke, limb ischemia, MI, renal artery thrombosis, and bowel ischemia) was detected in 12 (6.9%) (Table 1).

**Table 1:** Frequency and sort of the thromboembolic events associated with our COVID-19 patients

[DVT] Deep venous thromboembolism no. (%)	2 (1.2)
[PE] Pulmonary embolism no. (%)	48(27.7)
PE + DVT no. (%)	3(1.7)
Renal artery thromboembolism no. (%)	1(0.5)
Stroke no. (%)	6 (3.5)
Acute coronary syndrome no. (%)	2(1.2)
Mesenteric/bowel ischemia no. (%)	2(1.2)
Limb ischemia no. (%)	1(0.5)
No thrombosis no. (%)	108 (62.4)
Total no. (%)	**173 (100)**

The two groups showed comparable baseline demographic characteristics except for age and smoking which were significantly higher in patients with thrombosis (*p* < 0.001). There was no statistically significant difference between the two groups in the terms of gender distribution, nationality, and reported comorbidities (*p* > 0.05). Signs, symptoms, and outcome measures were also comparable in both groups, except for chest pain which was significantly higher in patients complicated by thrombosis, for further information on the baseline characteristics refer to (Table 2).

**Table 2:** Baseline demographic, comorbidities, and associated diseases in two groups of COVID patients

Demographic variable	Patients with thrombotic events (*n* = 65)	Patients without thrombotic events (*n* = 108)	*P* value
Demographics			
Age (years)	57.46(12.03)	50.87(14.76)	0.002*^1^
Gender			
Male Female	49(75.4) 16(24.6)	71(65.7) 37(34.3)	0.183^2^
Nationality			
Saudi Non-Saudi	26(40) 39(60)	48(44.4) 60(55.6)	0.567^2^
BMI (kg/m^2^)	27.36((23.5–31.2)	26.02(24.04–31.23)	0.944^3^
Smoking			
No smoking	23(35.4)	64(59.3)	
Smoker Unknown	39(60.0) 3(4.6)	29(26.9) 15(13.9)	< 0.001*^2^
Comorbidities no. (%)			
Diabetes mellitus	33(50.8)	43(39.8)	0.160^2^
Hypertension	29(44.6)	38(35.2)	0.218^2^
Ischemic heart disease	16(24.6)	17(15.7)	0.150^2^
Heart failure	6(9.2)	6(5.6)	0.370^2^
Renal failure	4(6.2)	7(6.5)	1.000^2^
Malignancy	1(1.5)	0	NA
Sickle cell disease	0	3(2.8)	NA
HIV	0	1(0.9)	NA
Pulmonary disease (other than pulmonary embolism) no. (%)			
No	62(95.4)	90(83.3)	
COPD	1(1.5)	7(6.5)	
Asthma Tuberculosis IPF Pulmonary HTN	1(1.5) 0 0 1(1.5)	8(7.4) 2(1.9) 1(0.9) 0	0.110^2^
Sign and symptoms (at presentation to hospital) no. (%)			
Fever	50(76.9)	80(74.1)	0.675^2^
Cough	54(83.1)	85(78.7)	0.483^2^
Sore throat	24(36.9)	47(43.5)	0.393^2^
Dyspnea	58(89.2)	98(90.7)	0.747^2^
Hemoptysis	7(10.8)	11(10.2)	0.903^2^
Chest pain	25(38.5)	26(24.1)	0.044*^2^
Vomiting	12(18.5)	28(25.9)	0.259^2^
Diarrhea	16(24.6)	33(30.6)	0.401^2^
Nausea	22(33.8)	39(36.1)	0.763^2^
Loss of smell	6(9.2)	18(16.7)	0.171^2^
Loss of taste	8(12.3)	17(15.7)	0.534^2^
Headache	28(43.1)	42(38.9)	0.587^2^
Bone ache	37(56.9)	64(59.3)	0.763^2^
Limb weakness	4(6.15)	0	NA
Calf pain	4(6.15)	0	NA
Abdominal tenderness	2(3.08)	0	NA
Other no. (%)			
Duration of stay at hospital (days)	16(10–23)	14(10–20.75)	0.468^3^
Duration between admission and suspicion of thrombosis	7(3–10)	5(3–9.75)	0.509^3^
Outcomes (survival) no. (%)			
Survived Died	47(72.3) 18(27.7)	74(68.5) 34(31.5)	0.599^2^
ICU admission (yes)	51(78.5)	80(74.1)	0.515^2^
Intubation (yes)	39(60.0)	60(55.6)	0.567^2^
Duration of stay at the ICU	6(1–11)	5.5(0-13)	0.758^3^

Values are the mean and SD, median (IQR) or *n* (%), *BMI* body mass index, kg/m^2^ mean (SD), *HIV* human immunodeficiency virus, *COPD* chronic obstructive pulmonary disease, *IPF* idiopathic pulmonary fibrosis, *HTN* hypertension. *ICU* intensive care unit, *NA* not applicable

^1^Independent-sample *t* test

^2^Chi-square test

^3^Mann-Whitney test

Both groups were comparable in their vital sign measurements (respiratory rate, heart rate, oxygen saturation) and various blood test measurements (HGB, WBCs, INR, PT, PTT, and platelet count). However, D-dimer measures were significantly higher in patients with thrombotic complications.

There was no significant difference in CT parenchymal findings between both groups. Regarding ECHO findings, RV dilatation, and dysfunction were significantly higher in patients with thrombosis.

Except for aB2GPI (IgG), which was significantly higher in the group with thrombotic events, there was no significant difference between the two groups in the proportion of patients positive for aPLs (LAC, aCL, or a2GPI IgM) (*p* > 0.05) (Table 3).

**Table 3:** Vital signs, blood tests, radiological finding, ECHO findings, and frequency of positive aPLs in both groups at the time of suspicion of thrombosis

Variable	Patients with thrombotic events (***n*** = 65)	Patients without thrombotic events (***n*** = 108)	***P***
**Respiratory rate**	30(26–34.5)	29(25.25–32)	0.429^3^
**Heart rate**(b/m)	110(100–122)	109(100–117.8)	0.285^3^
**Oxygen saturation**%	88(80–90)	88(82–90)	0.834^3^
**HGB**(g/L),	124.48(22.2)	123.23(23.53)	0.731^1^
**WBCs**(10^9^/L)	11.27(7.3–14.4)	9.5(6.8–13.8)	0.225^3^
**Platelet count** (10^9^/L	253(187.5–336.5)	256.5(194–339)	0.909^3^
**PTT** (s)	34.8(29.6–39.35)	34.3(30.7–38.8)	0.614^3^
**PT** (s)	13.4(12.1–14.85)	13.05(12.3–14.6)	0.943^3^
**INR**	1.14(0.2)	1.11(0.2559)	0.085^1^
**D-dimer**(mg/L)	12.99(6.8–21.9)	3.68(2.05–7.2)	< 0.001*^3^
**CT parenchymal findings no. (%)**			
No pulmonary infiltrate Bilateral peripheral ground glass	2(3.1) 33(50.8)	50(46.3 50(46.3)	0.179^2^
Bilateral peripheral ground glass with consolidation	28(43.1)	8(7.4)	
Unilateral peripheral ground glass	2(3.1)	0	
**ECHO no. (%)**			
Not done	10(15.4)	17(15.7)	
Normal	10(15.4)	19(17.6)	
VRV dilatation or dysfunction Left ventricular dysfunction Pulmonary HTN	25(38.5) 6(9.2) 14(21.5)	19(17.6) 23(21.3) 30(27.8)	0.018*^2^
**Positive aPLs no. (%)**			
LAC	30(46.2)	44(40.7)	0.486^2^
aCL IgG aCL IgM	10(15.4) 4(6.2)	14(13) 5(4.6)	0.655^2^ 0.730^2^
aβ2GPI IgG aβ2GPI IgM	10(15.4) 6(9.2)	3(2.8) 3(2.8)	0.005*^2^ 0.082^2^
Any positive aPLs	35(53.8%)	53 (49%)	0.543^2^

*HGB* hemoglobin, *WBCs* white blood cells, *PTT* partial thromboplastin time, *PT* prothrombin time, *INR* international normalized ratio*. CT* computerized tomography, *ECHO* echocardiogram, *HTN* hypertension, *aPLs* anti-phospholipid antibodies, *LAC* lupus anti-coagulant*, aCL* anti-cardiolipin, *aβ2GPI* anti-β2 glycoprotein

*P<0.05 (significant value)

^1^Independent-sample *t* test

^2^Chi-square test

^3^Mann-Whitney test

Pulmonary embolism had the most common prevalence of all thrombotic events, 48 patients out of 65(73.8%). Among the various aPLs tested in thrombotic patients, lupus anti-coagulant (LAC) had the highest positivity (46.2 %). The presence of any circulating aCL (IgM) or aCL (IgG) has been found in 14 patients (~ 21.54%). The presence of any circulating aβ2GPI (IgM) and aβ2GPI (IgG) has been found in 16 (~ 24.62%) patients. Most patients with arterial thromboembolism (stroke, MI, limb ischemia, bowel ischemia, and renal artery thrombosis) had triple positivity of anti-phospholipid antibodies (Table 4).

**Table 4:** aPLs in different types of thrombosis in COVID-19 patients (*n* = 65)

Positive aPLs	DVT + PE 3 (4.6%)	PE 48(73.8%)	DVT 2(3.1%)	Stroke 6(9.2%)	Limb ischemia 1 (1.5%)	MI 2(3.1%)	RAT 1(1.5%)	Bowel ischemia 2 (3.1%)
LAC	1(33.3)	16(33.3)	2(100)	5(83.3)	1(100)	2(100)	1(100)	2(100)
aCL IgG aCL IgM	0(0) 0(0)	5(10.4) 3(6.3)	0(0) 0(0)	2(33.3) 1(16.7)	0(0) 0(0)	1(50) 0(0)	1(100) 0(0)	1(50) 0(0)
aβ2GPI gG aβ2GPI gM	0(0) 0(0)	6(12.5) 1(2.1)	1(50) 0(0)	2(33.3) 2(33.3)	0(0) 0(0)	1(50) 1(50)	1(100) 0(0)	1(50) 0(0)

*aPLs* anti-phospholipid antibodies, *LAC* lupus anti-coagulant, *aCL* anti-cardiolipin, *aβ2GPI* anti-β2 glycoprotein, *MI* myocardial infarction, *RAT* renal artery thrombosis, data are expressed as no. (%)

In COVID-19 patients with thrombotic consequences, there was no statistically significant difference in the positivity of aPLs (LAC, aCL (IgG–IgM), or aβ2GPI (IgG–IgM) between survivors and those who died (Table 5).

**Table 5:** aPLs and survival in COVID-19 patients with thrombotic events

Positive aPLs	Survived(*n* = 47)	Died(*n* = 18)	*P*
LAC	19(40.4)	11(61.1)	0.134^2^
aCL IgG aCL IgM	6(12.8) 3(6.4)	4(22.2) 1(5.6)	0.445^2^ 1.000^2^
aβ2GPI IgG aβ2GPI IgM	8(17.0) 5(10.6)	2(11.1) 1(5.6)	0.713^2^ 1.000^2^

*aPLs* anti-phospholipid antibodies, *LAC* lupus anti-coagulant, *aCL* anti-cardiolipin, *aβ2GPI* anti-β2 glycoprotein*,* data are expressed as no. (%)

^2^Chi square test

After 12 weeks, the number of positive LAC and aB2GPI IgM was significantly reduced (*p* = 0.005); however, there was no statistically significant difference between the two groups in aCL IgG and aCL IgM (Table 6).

**Table 6:** Comparing aPLs during hospital admission and after 12 weeks of the first sample in survived positive patients (any positive aPLs test) with thrombotic events (*N* = 23)

*aPLs*	During admission (*n* = 23)	After 12 weeks (*n* = 23)	*P*
LAC			
Positive Negative	19(82.6) 4(17.4)	1(4.3) 22(95.7)	< 0.001*^4^
aβ2GPI IgG			
Positive	8(34.8)	1(4.3)	0.016*^4^
Negative	15(65.2)	22(95.7)	
aβ2GPI IgM			
Positive Negative	5(21.7) 18(78.3)	1(4.3) 22(95.7)	0.219^4^
aCL IgG			
Positive	6(26.1)	1(4.3)	0.125^4^
Negative	17(73.9)	22(95.7)	
aCL IgM			
Positive Negative	3(13) 20(87)	0(0) 23(100)	0.250^4^

*aPLs* anti-phospholipid antibodies, *LAC* lupus anti-coagulant, *aCL* anti-cardiolipin, *aβ2GPI* anti-β2 glycoprotein, *NA* not applicable, *data are expressed as no. (%)*

*P<0.05 (significant value)

^4^McNemar test

## Discussion

Since the emergence of the COVID-19 pandemic, serious thrombotic consequences have been reported in infected patients, especially those who are critically ill [4]. Even with prophylactic or therapeutic anti-coagulation, COVID-19 patients experienced a higher-than-expected number of thrombotic episodes, both venous (pulmonary thromboembolism, venous sinus thrombosis, deep vein thrombosis) and arterial (myocardial infarction and stroke) [4].

In our present study, venous thrombosis includes (PE, DVT, and PE + DVT) was detected in 53 (30.6%) out of 173 patients; however, arterial thrombosis includes (stroke, limb ischemia, MI, renal artery thrombosis, and bowel ischemia) was detected in 12 (6.9%). This agreed with a recent meta-analysis [16] of 42 trials, including 8271 COVID-19 patients found an overall VTE incidence was 21%, with a DVT rate of 20% and PE rate of 13%, whereas the ATE rate was 2%. In critically ill patients, the VTE rate was 31% and ATE rate was 5%.

Our data revealed that pulmonary embolism was the most common thrombotic consequence, with PE occurring in 51 of 65 (73.8%) patients who had thrombotic episodes. These findings are consistent with Klok et al. findings of a high incidence of VTE (31%) leading to complications such as PE (80%) [4]. In severe COVID-19 pneumonia patients, the high prevalence of PE has been explained by the inflammatory nature of the disease rather than by an embolic mechanism of DVT [17].

Infections with COVID-19 may cause macrovascular and microvascular thrombosis through a variety of synergistic mechanisms. For example, a cytokine storm activates leukocytes, platelets, endothelium, hypoxic vaso-occlusion, and virus infection that directly activates immune and vascular cells [18]. COVID-19 is unique because it directly infects vascular endothelial cells; this dysfunction appears to be a critical signal for thrombosis [18]. Furthermore, the prevalence of thrombotic stroke, especially in young individuals, gives some clinical evidence that aPLs may be involved in endothelial dysfunction [11, 12]. Our demographic characteristics agree with that except for the age; older age showed a significantly increased risk of developing thrombosis.

Like our results, multiple reports documented increased D-dimer levels [8, 19, 20] associated with severe infection [21]. D-dimer levels seem a prognostic indicator as they increased to be 4-fold higher in patients who did not survive than survivors [8].

A recent study [14] demonstrated about one out of every two COVID-19 patients were tested positive for LAC, but aCL and a2GPI antibodies are less common (around 10% for each). Among the various aPLs tested in our study, lupus anti-coagulant (LAC) had the highest rate of positivity (46.2%) in the thrombotic group, whereas aCL (IgM or IgG) and anti-ß2 GPI (IgM or IgG) were about 21.6 and 24.6%, respectively. This agreed with a recent meta-analysis involving 1159 patients (from 21 studies) admitted with COVID-19. The most frequent aPL detected was LAC, with pooled prevalence rate of 50.7% (95% CI 34.8 to 66.5%), whereas the prevalence rate of aCL (IgM or IgG) and anti-ß2 GPI (IgM or IgG) were 13.9% (95% CI 7.5 to 24.1%) and 6.7% (95% CI 3.5 to 12.5%), respectively [22].

The American Society of Hematology (ASH) performed an anti-phospholipid antibody (aPLs) testing. Only 4 out of 27 cases with COVID-19 exhibited lupus anti-coagulant (LAC) [23]. On the other hand, no patients tested positive for anti-aCL or anti-a2GPI antibodies. Nevertheless, the ASH strongly advised against routine aPLs testing in COVID-19 cases unless clinically recommended by the history or for a study protocol [24] due to the well-known fact that aPLs might occur transiently after acute infection, inflammation, or thrombosis. Other ambivalent researchers have investigated the prothrombotic effects of aPLs and come up with conflicting conclusions. aPLs have been linked with the development of arterial thrombosis, notably pulmonary embolism and stroke, in several investigations [10, 25–29]. In conjunction with this research, this study suggests that COVID-19 infection increases the likelihood of acquiring aPLs. Although aPLs can alter hemostatic systems to cause thrombotic events, their existence in COVID-19 patients is not always accompanied by a thrombotic event.

A recent study has documented that aPLs, even in mild or transitory titers, are commonly present in hospitalized patients for COVID-19 [30]. Evidence in the literature has shown that patients with greater than one positive test, particularly those with triple positivity (LAC, aCL, and aβ2GPI), have an increased risk of thrombotic APS [30]. Double positivity (mostly LAC negative) is generally at lower thrombotic risk [31]. The reliability of the results was affected by the presence of antibodies of the same isotype [32]. Patients with isolated positive LAC, but negative aCL and aβ2GPI, have a low risk of a thromboembolic event [33]. In agreement with that, 13.8% of our thrombotic patients had triple positivity of (LAC, anti-cardiolipin, and anti-β2 glycoprotein I) antibodies. Also, we demonstrated that patients with arterial thrombosis (stroke, MI, limb ischemia, bowel ischemia, and renal artery thrombosis) had triple positivity of anti-phospholipid antibodies.

Although the presence of aPLs is characteristic of many infections, their occurrence does not always imply the development of thrombotic complications and, consequently, the anti-phospholipid syndrome (APS) [33]. The frequency of aPL antibodies involving a healthy population is demonstrated in studies with relatively low percentages, e.g., in a healthy control cohort of 200 people, IgG/IgM/IgA aCL 1%/1%/3%, and IgG/IgM/IgA anti-β2GPI 4%/1%/1% showed elevated levels [34]. Another study found that the prevalence of aPLs in the healthy population ranged from 1 to 5.6% [35]. In severe COVID-19, aPLs (aCL and a2GPI Ig) increase, but not in mild cases, suggesting that a vigorous anti-viral immunoglobulin response, potentially initiated in the bronchial mucosa, may cause systemic autoimmunity [26].

The Subcommittee for the Standardization (SCC) for LA and aPLs of the International Society of Thrombosis and Hemostasis (ISTH), in its latest update, endorses testing all three tests (LAC, aCL, and a2GPI) to detect APS-related thrombosis and should also validate positive laboratory findings 12 weeks following the original assessment [30]. Re-testing after 3 months is indicated to ensure reliability, especially in cases of an initial triple-positive test [30].

In our study, we retested aPLs for only 23 patients after 12 weeks from the initial sample and found that 2 out of 23 previously positive patients for aPLs were positive after 12 weeks. This finding is consistent with a previous study that found a reduction in aPLs positivity (9 patients out of 10 were negative) after 1 month follow-up [36], raising the fact that these aPLs increase transiently, as an inflammatory-mediated condition, and do not remain high enough to meet current APS classification requirements.

We found no significant differences in aPLs positivity between survived and dead patients, which is consistent with previous research [37], which found no significant link between aPLs positivity and mortality in COVID-19 patients with thrombotic complications. This could be explained by the associated severe pneumonia, which is the leading cause of death in COVID-19 patients.

Our study is limited by the small number of cases included (single-center study). Therefore, it is essential to conduct further studies that specifically test aPL antibodies in a larger context to make subsequent important statements about the role of APS in COVID-19 and further strengthen the significance of the described comparisons.

Also, the assessment of LAC in our study was challenged using unfractionated heparin and low molecular weight heparin (LWMH) that can lead to false-positive results. Anti-phospholipid antibodies (aCL and aβ2GPI) detection is tiresome. There are several commercial assays, and even for the identical assays, inter-laboratory variability is considerable [38]. Furthermore, ELISA test results for aCL and 2GPI should be regarded positive if they are higher than the cut-off value, which is determined as more than the 99th percentile [39].

## Conclusions

Our study revealed a high incidence of thrombotic consequences in severe COVID-19 pneumonia cases. This study supports an increased risk of developing aPLs following COVID-19 infection. Although aPLs can modify the hemostatic mechanisms towards thrombotic phenomena, their presence is not always accompanied by a thrombotic event in COVID-19 patients. These aPLs increased transiently as an inflammatory-mediated condition and did not remain high enough to meet current APS classification requirements. Individuals with aPLs triple positivity had a marked risk of arterial thrombosis. Also, we did not detect significant differences between survived and non-survived patients regarding the positivity of aPLs. Therefore, we did not support screening COVID-19 patients for aPL by evidence.

## Data Availability

The data will be available upon reasonable request.

## Abbreviations

COVID-19: Coronavirus disease 2019
SARS-CoV-2: Severe acute respiratory syndrome coronavirus 2
VTE: Venous thromboembolism
PE: Pulmonary embolism
DVT: Deep vein thrombosis
ATE: Arterial thrombosis
IL: Interleukin
TNF: Tumor necrosis factor
aPLs: Anti-phospholipid antibodies
aCL: Anti-cardiolipin
LAC: Lupus anti-coagulant
aB2GPI: Anti-B2-glycoprotein I
HTLV-1: Human T-lymphotropic virus type 1
EBV: Epstein-Barr virus
CMV: Cytomegalovirus
DIC: Disseminated intravascular coagulation
CTPA: Computed tomography pulmonary angiogram
